# Isolated posterior stabilization in type B and C thoracolumbar fractures associated with ankylosing spine disorders: A single center experience with clinical and radiological outcomes

**DOI:** 10.1051/sicotj/2024022

**Published:** 2024-08-09

**Authors:** Benoit Sulpis, Thomas Neri, Antonio Klasan, Xavier Castel, François Vassal, Marie Charlotte Tetard

**Affiliations:** 1 Jacques Lisfranc Faculty of Medicine, Jean Monnet University 10 Rue de la Marandière 42270 Saint-Priest-en-Jarez France; 2 Department of Neuro Surgery, University Hospital of Saint Etienne Hôpital Nord 42055 Saint-Étienne Cedex 2 France; 3 Department of Orthopaedic Surgery, University Hospital of Saint Etienne Saint Etienne France; 4 EA 7424 - Inter-University Laboratory of Human Movement Science, University of Lyon - Jean Monnet University Saint-Étienne Cedex 2 France; 5 Department for Orthopedics and Traumatology, Kepler University Hospital GmbH Krankenhausstrasse 9 4020 Linz Austria; 6 Johannes Kepler University Linz Altenberger Strasse 69 4040 Linz Austria

**Keywords:** Ankylosing spine disorders, Percutaneous, Posterior stabilization, Thoraco-lumbar fracture

## Abstract

*Introduction*: Fractures in ankylosing spine disorders (ASD) are associated with high complication and mortality rates. During the posterior stabilization of these fractures, reduction is often partial, resulting in the persistence of a significant anterior diastasis. Our objective was to evaluate the safety and efficiency of isolated posterior stabilization in elderly ASD patients, without direct reduction of the anterior diastasis, in terms of clinical and radiological outcomes, complications, and mortality. *Methods*: This retrospective study included 46 patients, mean age 79.3 years, with ASD, who underwent isolated posterior stabilization, open or percutaneous, for thoracolumbar fractures. The average follow-up was 21.7 months, with a minimum follow-up of 6 months. Autonomy (Parker score) and radiological results (lordotic angulation) were analyzed pre-and post-operatively. *Results*: Autonomy was maintained at the last follow-up, with no significant difference in Parker’s score. The consolidation rate was 94.6%. No implant failure was recorded. Despite the absence of an anterior procedure, lordotic angulation was significantly reduced by 2.6° at 6 months (*p* = 0.02). The rate of surgical complications following open surgeries was 10.9% (*n* = 5), of which 6.5% were infections. No surgical complications were reported in percutaneous surgeries. The rate of medical complications was 67.4% (*n* = 31), with a rate of 88.2% in the open surgery group, compared to 55.2% in the percutaneous surgery group. An open approach was associated with a five-fold higher risk of complications (*p* = 0.049). Nine patients died during follow-up (19.6%). *Conclusions*: Isolated posterior stabilization in the treatment of thoracolumbar spine fractures in elderly ASD patients is a safe technique promoting autonomy preservation, and high radiological bony healing with acceptable complication and mortality rates. The persistent anterior gap is partially reduced when the spine is loaded and does not seem to require an anterior procedure, thus decreasing complications. Percutaneous surgery should be the technique of choice to reduce surgical complications.

## Introduction

Ankylosing spondylitis (AS) and diffuse idiopathic skeletal hyperostosis (DISH) are the most common ankylosing spinal disorders (ASD). AS is a chronic inflammatory autoimmune joint disease involving the axial skeleton often leading to ankylosis [1]. DISH is a non-inflammatory disease defined by anterolateral ossification of the anterior longitudinal ligament [2]. Its etiology is unknown, but it has been associated with various metabolic disorders, including obesity and insulin-dependent diabetes [3].

ASD patients face a particularly elevated risk of spinal fractures, with 3 to 4-fold higher risk in AS compared to a population without ASD [4]. Moreover, the nature of these fractures differs significantly, with the majority classified as distraction fractures (type B) or type C (according to the AOSpine Thoracolumbar Spine Injury Classification System). Pathological vertebral remodeling, spinal rigidity, and osteopenia commonly associated with ASD, decrease the spine’s resistance to impacts, explaining the occurrence of these fractures [5]. Consequently, even minor traumas can lead to complex and unstable fractures.

The highly unstable nature of these fractures requires surgical stabilization in order to reduce morbidity and mortality [6, 7] and promote bone healing. Mortality rates for fractures in ASD patients exceed those observed in the general spinal trauma population. In the Westerveld et al. series, intra-hospital mortality was 9.5% for surgically treated DISH patients compared to 2.7% for control patients [7]. Stabilization is usually achieved by dorsal instrumentation, open or percutaneous. In recent years, the development of percutaneous fixation techniques has proven effective in reducing postoperative complications, especially in frail patients [8].

In hyperextension fractures of an ankylosed spine, the anterior fracture gap is further widened when the patient is placed in a prone position during surgery. Some authors describe additional anterior fusion [9]. While it can allow for better fracture reduction and alignment restoration, anterior fusion is an additional procedure and is associated with increased risks of complications, as high as 19% [10].

The majority of the studies on ASD focus on patient and fracture characteristics and complication or mortality rates. To our knowledge, only a few have studied the patient’s functional recovery and restoration of sagittal balance with posterior-only instrumentation. Lindtner et al. achieved good fracture reduction with less rigid rods and post-operative mobilization, but without assessing the mortality rate and radiological healing [11]. Kohler et al. compared fracture reduction between open surgery and minimally invasive surgery groups, without specifically examining autonomy and bone healing [12].

We hypothesized was that in thoracolumbar fractures in ASD, isolated posterior stabilization, was a safe and efficient technique, allowing satisfying outcomes in terms of sagittal alignment restoration, without the need for open reduction of the fracture lordotic angulation, and self-sufficiency preservation, with acceptable complication and mortality rates. Our primary objective was to evaluate clinical and radiological results. Our secondary objective was to assess complication and mortality rates.

## Material and methods

It was a retrospective, observational, monocentric study. Between December 2017 and November 2020, all patients over 65 years of age admitted to the neurosurgery department of the Saint Etienne University Hospital Center for a thoracolumbar fracture managed by open or percutaneous instrumentation and suffering from ASD of DISH or AS type were included. The DISH was defined according to Resnick’s radiological criteria [2].

The choice between percutaneous pedicle screwing or the conventional open posterior fixation depended on the visibility of the pedicles with the C-Arm or the need to perform decompression.

In total, 46 patients were included with an average follow-up of 21.7 ± 13.1 months (including early deaths). The fractures were mainly located in the thoracic spine (*n* = 36, 78.3%), T11 being the most frequently affected level (15.2%). In 93.5% of cases, the fractures were type B (*n* = 43). Three fractures were type C. The time from trauma to surgery was due to a delay in diagnosis in 10 cases (21.7%), including a delay in consultation in 3 cases, anticoagulant or anti-platelet treatment (*n* = 17), and treatment of medical complications before surgery (*n* = 11). On admission, 15.2% of patients had a neurological deficit. The characteristics of the included population are recorded in Table 1.

**Table 1:** Baseline characteristics of the 46 study patients.

Parameter	*n* = 46 patients
**Sex**	
M	30 (65.2%)
F	16 (34.8%)
**Age (years), Mean ± SD (range)**	79.3 ± 7 [68; 95]
**BMI, Mean ± SD (range)**	28.8 ± 5.6 [20.2–41.1]
**Etiologies**	
DISH	44 (95.7%)
SA	2 (4.3%)
**ASA score**	
ASA 2	*n* = 21 (45.7%)
ASA 3	*n* = 19 (41.3%)
ASA 4	*n* = 6 (13%)
**Comorbidities**	
Cardiovascular	19 (41.3%)
Pulmonary	12 (26.1%)
Diabetes	13 (28.3%)
Neuromuscular	8 (17.4%)
Renal	6 (13%)
Malignancy	10 (21.7%)
**ASIA score**	
A	*n* = 2 (4.3%)
B	*n* = 1 (2.2%)
C	*n* = 4 (8.7%)
E	*n* = 39 (84.8%)
**Mean time to surgery (days)**	8.6 ± 6.4 [0; 27]
**Baseline autonomy (Parker score)**	
0–3	5 (10.9%)
4–6	6 (13%)
7–9	35 (76.1%)
**Trauma mechanism**	
High-energy, polytrauma	*n* = 21 (45.7%)
Low-energy (ground-level fall)	*n* = 25 (54.3%)
Associated traumas	18 (39.1%)
Polytrauma	6 (13%)
Epidural hematoma with neurological impairment	3 (6.5%)
Associated vertebral fractures	9 (19.6%)
**Length of stay (days), Mean ± SD (range)**	19.1 ± 18.3 [5; 124]
**Follow-up (months), Mean ± SD (range)**	21.7 ± 13.1 [1; 53]

BMI: body mass index; DISH: diffuse idiopathic skeletal hyperostosis; AS: ankylosing spondylitis; ASA: American Society of Anesthesiologists.

**Figure 1:**
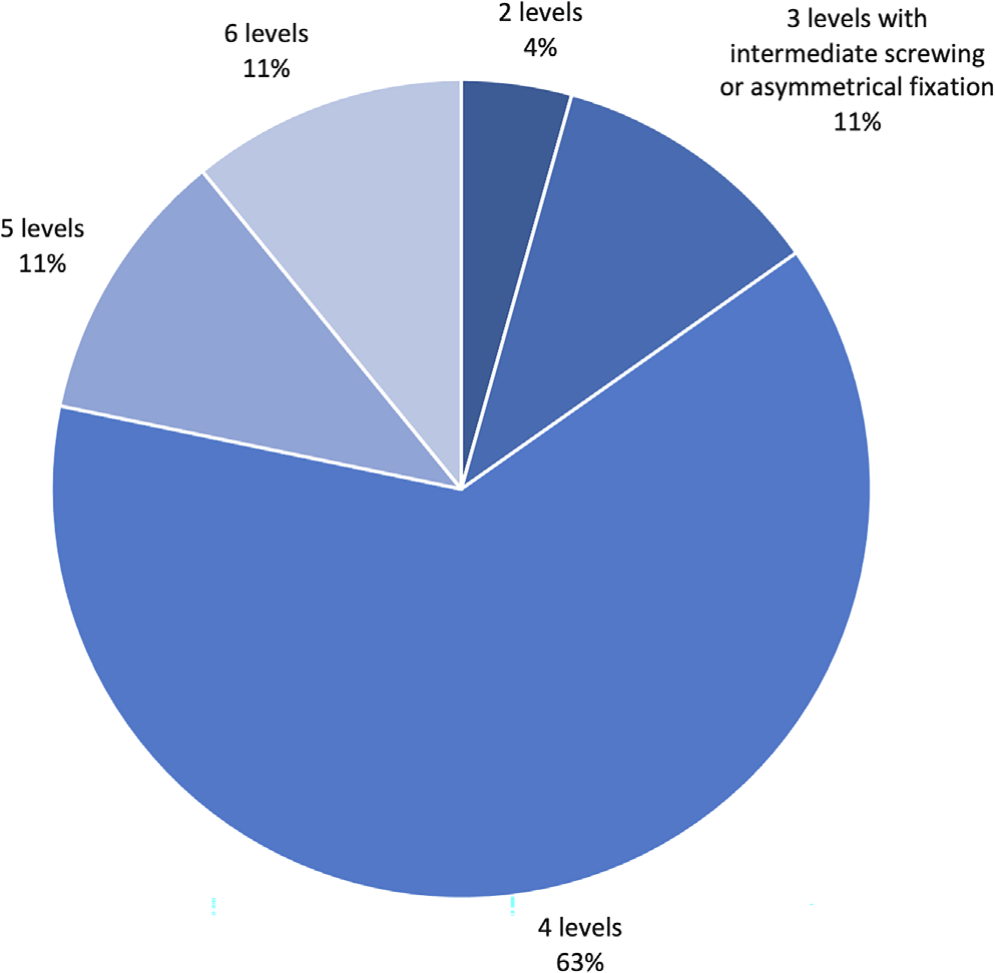
Number of instrumented levels per patient, showing a predominance of 4-level fixation.

Seventeen patients (37%) were treated by open internal fixation and 29 patients were treated by percutaneous instrumentation (63%). Screw cementation was performed in cases of poor bone quality (*n* = 30, 65.2%), which was decided based on the radiological signs of osteoporosis and/or during surgery. In these patients, all the screws were cemented. The number of instrumented levels per patient was distributed as shown in Figure 1. Decompression by laminectomy was performed in 6 cases (13%).

Clinical and radiological follow-up was performed immediately before and after the operation (during in-hospital stay) and then at last follow-up, with a minimum of 6 months post-operatively. The American Society of Anesthesiologists (ASA) score was obtained from the pre-operative anesthesia consultation.

Clinical parameters recorded were the following: neurological status on admission according to the ASIA score [13], intra- and post-operative medical and surgical complications, mortality, baseline autonomy, and at last follow-up using the Parker score. The Parker Mobility Score rates three aspects of mobility using a 0–3 scale: moving within the house, leaving the house, and shopping. Scores range from 3 (no difficulty) to 0 (no mobility), with 9 being the best possible overall score [14].

**Figure 2:**
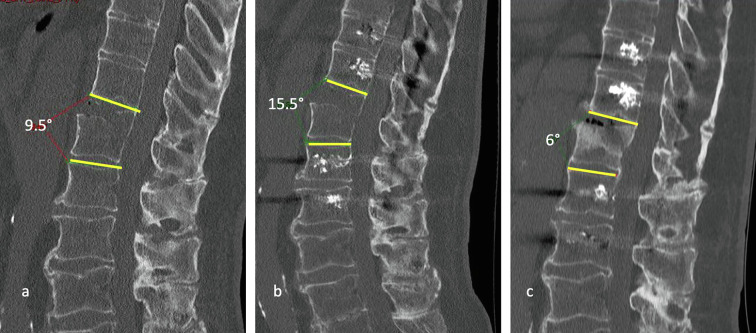
CT scan of a 75-year-old patient in the pre-operative (a), immediate post-operative (b), and 6 months post-operative (c) stages, who underwent cemented percutaneous long posterior instrumentation for a T11–T12 fracture. Despite the absence of an anterior procedure, there is a progressive correction of the anterior diastasis at 6 months: preoperative lordotic angulation was 9.5°, immediate postoperative angulation was 15.5° and angulation at the late postoperative stage was 6°.

The radiological evaluation involving 37 patients (3 lost to follow-up, 6 early deaths), was made on computerized tomography (CT) scans. In some cases, an additional MRI scan was useful to specify the type of fracture (disc fracture, posterior ligament rupture), and to identify associated lesions such as an epidural hematoma. The immediate post-operative CT scan allowed visualization of the pedicle screw position and the reduction of the fracture. At 6 months, a CT scan was performed in order to evaluate fracture healing and/or implant loosening. Lordotic angulation (reflecting the anterior diastasis) was calculated on a true sagittal reconstruction, as shown in Figure 2, at pre-operative, immediate postoperative, and last radiological follow-up stages. In type C fractures, it was not possible to calculate the angulation in a reproducible manner.

The statistical tests were done with the software SPSS Statistics version 28.0.1 (IBM^®^, Armonk, NY, USA). The significance threshold was set at 5%. All tests were bilateral.

Patient characteristics were described as frequencies and percentages for qualitative variables, and as mean ± standard deviation [range] for quantitative variables.

The relationships between variables were explored in univariate analysis using raw Odds Ratio (OR) with 95% confidence intervals, Fisher tests for qualitative variables, and Wilcoxon tests for quantitative variables. If quantitative variables were normally distributed, as assessed by Shapiro-Wilk’s test (*p* > 0.05), Student tests were performed.

## Results

**Figure 3:**
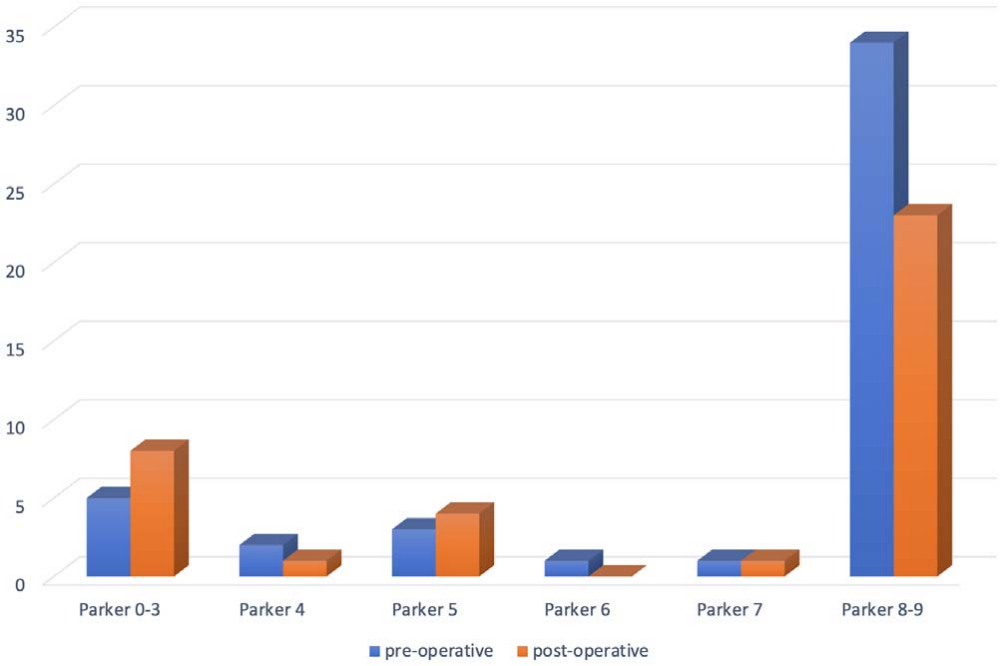
Distribution of Parker score preoperatively and postoperatively at last follow-up, showing a slight decrease in self-sufficiency. Twelve patients (32.4%) experienced a loss of one or more points on the Parker score.

No significative difference was seen in the pre- vs post-operative Parker Mobility Score (7.59 vs 6.59, *p* = 0.142). However, autonomy at the last follow-up was slightly reduced in comparison with the preoperative period: from 75.7% to 62.2% of Parker 9 patients (excluding deaths) and 12 patients (32.4%) with a loss of one or more points on the Parker score (Figure 3). Returning to the previous accommodation setting at the last follow-up was possible in 86.5% of the cases (*n* = 32, excluding deaths). Age, female gender, pre-fracture level of autonomy, pre-operative neurological impairment, and complications (all combined) were correlated with loss of autonomy (Table 2).

**Table 2:** Predictive factors for mortality, postoperative complications, and loss of autonomy.

Factors	Mortality		Complications		Loss of autonomy	
	OR (IC 95%)	*p*-value	OR (IC 95%)	*p*-value	OR (IC 95%)	*p*-value
Age	1.12 (1–1.25)	*p* = 0.042*	1.22 (1.06–1.4)	*p* < 0.001*	1.15 (1.04–1.28)	*p* = 0.003*
Sex	Male: 1.08 (0.23–5.06)	*p* = 1	Male: 0,40 (0.09–1.71)	*p* = 0.316	Female: 7.00 (1.77–27.68)	*p* = 0.005*
Time to surgery	1.03 (0.93–1.15)	*p* = 0.55	0.999 (0.905–1.102)	*p* = 0.981	0.96 (0.88–1.06)	*p* = 0.433
BMI	0.93 (0.8–1.07)	*p* = 0.283	1.05 (0.93–1.18)	*p* = 0.423	1.03 (0.93–1.15)	*p* = 0.526
ASA score	1.06 (0.25–4.60)	*p* = 1.00	8.07 (1.84–35.41)	*p* = 0.004*	2.55 (0.77–8.48)	*p* = 0.149
Baseline autonomy	2.14 (0.43–10.74)	*p* = 0.384	5.09 (0.58–44.78)	*p* = 0.143	7.08 (1.30–38.44)	*p* = 0.028*
Associated trauma	21.6 (2.39–195.3)	*p* = 0.001*	6.00 (1.15–31.23)	*p* = 0.027*	2.83 (0.83–9.61)	*p* = 0.132
Open surgery	1.48 (0.34–6.47)	*p* = 0.707	5.29 (1.02–27.57)	*p* = 0.049*	2.34 (0.69–7.94)	*p* = 0.225
Trauma mechanism	0.61 (0.14–2.64)	*p* = 0.711	1.95 (0.55–6.95)	*p* = 0.349	2.55 (0.77–8.48)	*p* = 0.149
Initial neurological status	4.13 (0.73–23.3)	*p* = 0.124	—	*p* = 0.083	9,6 (1.05–87.78)	*p* = 0.036*
Complications	**—**	*p* = 0.041*	—	—	8.77 (1.68–45.88)	*p* = 0.009*

Data presented as odds ratio with 95% confidence interval.

*p ≤ 0.05.

BMI: body mass index; ASA: American Society of Anesthesiologists.

**Figure 4:**
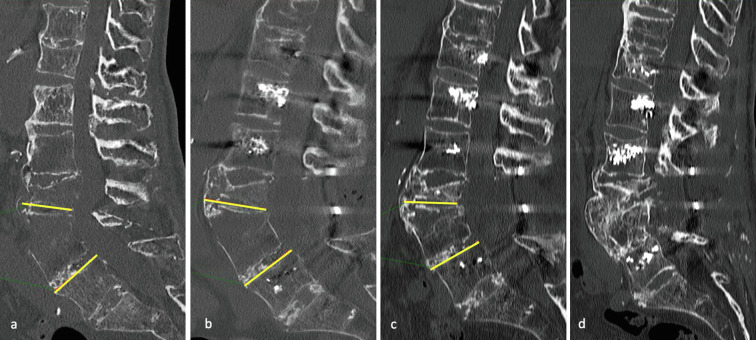
84-year-old female with a hyperextension fracture of L4, treated by open posterior cemented osteosynthesis (radicular decompression associated), with 47° of anterior diastasis in the pre-operative CT scan (a). Immediate post-operative CT scan showing no relevant reduction of the anterior diastasis measured at 43° (b). At 6 months, the CT scan shows a significant reduction to a 30° anterior diastasis (c), allowing for consolidation through anterolateral osseous bridging (d).

**Figure 5:**
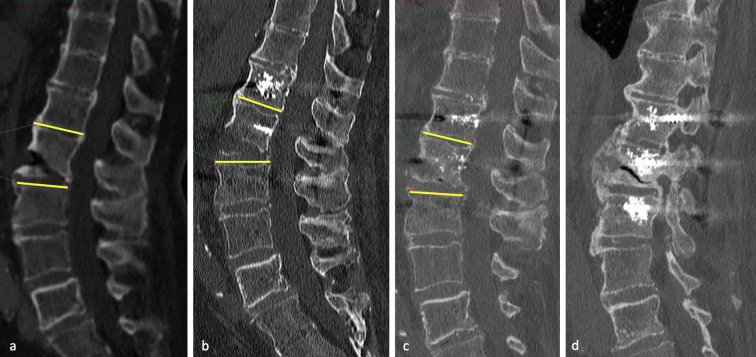
Imaging of a 79-year-old female, who sustained a transcorporeal hyperextension fracture of L2, with 10° of anterior diastasis before surgery (a), treated by cemented percutaneous osteosynthesis. We observed an increase in anterior diastasis to 21° immediately after surgery (b), which reduced to 9° at the last follow-up (c), enabling osseous healing despite necrosis at the fracture site (d).

Radiological analysis at the last follow-up showed consolidation in 94.6% (*n* = 35) of cases. One case of complete non-union and one partial consolidation remained asymptomatic and did not require revision surgery. No implant loosening (for uncemented or cemented screws) or secondary fracture displacement was reported. Lordotic angulation was significant (≥5°) in 19 patients (41.3%). The mean lordotic angulation was 4.5 ± 11.4° preoperatively, 6.1 ± 11.6° in the immediate postoperative period, and 2 ± 8.2° at the last follow-up. The average correction in angulation from preoperative to the last follow-up was 2.6° (*p* = 0.02), and between the immediate postoperative period and last follow-up was 4.1° (*p* < 0.001) (Figures 2, 4, 5).

The rate of surgical complications was 10.9%. Three cases of deep surgical site infection (6.5%), all of them early (<3 months), required revision surgery. One patient had a recurrence of infection 2 years later requiring revision surgery with implant removal. The overall reoperation rate was 13%. The medical complications rate (before 3 months) was 67.4% (*n* = 31 patients) (Table 3). The ASA scores 3 and 4 were correlated with the occurrence of complications (OR = 8.1, *p* = 0.004). The open approach was associated with a five-fold higher risk of complications (*p* = 0.049) (Table 2).

**Table 3:** Main post-operative complications.

**Medical complications**	**31 patients (67.4%)**
Pulmonary complication	12 (26.1%)
Urinary tract infection	12 (26.1%)
Deep venous thrombosis/Pulmonary embolism	3 (6.5%)/2 (4.3%)
Confusional syndrome	4 (8.7%)
Bed-rest complications	5 (10.9%)
Digestive complications (gastric ulcer, peritonitis)	3 (6.5%)
Ischemic stroke	1 (2.2%)
Multi-visceral failure	1 (2.2%)
**Surgical complications**	**5 (10.9%)**
Surgical site infection	3 (6.5%)
Epidural hematoma	1 (2.2%)
Meningitis (traumatic dural tear)	1 (2.2%)

At the last follow-up, 9 patients died (19.6%), all over 75 years of age. Six deaths occurred in the first month after surgery and the others between 3 and 8 months after surgery. Six of the nine deaths (66.7%) were associated with severe pulmonary complications. In 3 out of 9 cases, a pre-operative neurological deficit was present. Risk factors for mortality are reported in Table 2.

## Discussion

Managing spinal fractures in patients with ASD is challenging due to patient fragility, instability caused by these fractures occurring on a brittle spine, and diagnostic difficulties. In this single-center cohort of elderly patients with ASD, isolated posterior stabilization in the treatment of thoracolumbar fractures showed good results in terms of functional recovery with no significative change in the Parker Mobility Score and return to previous conditions. Despite the absence of an anterior procedure, this posterior-only approach resulted in bone healing and reduction of anterior diastasis.

Our study had several limitations. The moderate size of the cohort contributed to a decrease in the power of our statistical analysis and we could not perform a multivariate analysis. However, due to the low incidence of thoracolumbar fractures in ASD, our population size remains comparable to the literature series [9, 15, 16]. A comparative study with a group combining anterior and posterior procedures could be even more informative, but technically extremely difficult to perform, especially in a controlled manner. In our department, the use of isolated posterior stabilization is widely practiced for this indication, and a control group with a more invasive procedure would pose a logistical and ethical challenge, given the results achieved with posterior-only instrumentation.

An anterior approach seems appealing in hyperextension injuries in ASD patients, allowing for direct fracture reduction. However, there are major pitfalls: the risk of implant failure due to osteoporosis or the high mechanical stress due to long-lever arms resulting from ankylosis, limited access due to thoracic kyphosis, and frequent perioperative pulmonary complications (more than a quarter of patients in our series) [11].

Posterior-only stabilization in ASD patients preserves autonomy, which is the main goal in elderly, frail patients. Parker’s score was maintained with only some patients experiencing a moderate decrease in self-sufficiency. In most cases, our patients could return to their previous accommodation setting. These results are in agreement with the literature, which showed no significative variation in Parker score [11, 15] and that quality of life was only slightly affected [15, 17] (Table 4).

**Table 4:** Table summarizing the results of main studies.

Studies	Mean age, *y*	Region of focus	Clinical outcomes	Radiological outcomes		Mortality
			Autonomy	Fracture healing	Implant loosening	
Backhaus et al. [18]	67	C/T/L	–	–	15%	–
Bernstein et al. [19]	70	C/T/L	–	–	–	23% at 1 year
Moussallem et al. [20]	75.56	T/L	–	–	–	5% at 2 months
Bredin et al. [15]	75.1	T/L	No significant difference in Parker’s score	100%	0.4% of screws	9.6% at 3 months
Westerveld et al. [7]	DISH: 68.4, AS: 69.5	C/T/L	–	100% of surgically treated	13.8%	In-hospital: 7.1% AS, 15% DISH
Vazan et al. [9]	73.4	C/T/L	–	–	–	In-hospital: 12.2% 39% at 1 year
Lindtner et al. [11]	74.7	T/L	No significant difference in Parker’s score	–	11.1% of patients	–
Ull et al. [21]	70.7	C/T/L	–	–	9.2%	In-hospital: 14.9%
Kohler et al. [12]	75	T/L	–	–	–	In-hospital: 0%
Our study	79.3	T/L	No significant difference in Parker’s score (loss of one or more points: 32.4%)	94.6% (no revision)	0%	19.6% at last follow-up

C: cervical; T: thoracic; L: lumbar; AS: ankylosing spondylitis; DISH: diffuse idiopathic skeletal hyperostosis.

Posterior instrumentation allows proper bone healing. Several authors have demonstrated this both in open [7, 22] and percutaneous surgeries [15–17]. Our findings support this trend (Table 4). The diffuse ossification process in ASD appears to facilitate the closure of the persistent anterior diastasis (Figures 4, 5).

Optimal intraoperative reduction of hyperextension lesions is often impossible to achieve due to the prone position. In the immediate postoperative period, a lordotic malalignment persists, often larger than in the pre-operative period (+1.6° on average). Posterior instrumentation counteracts reasonably well translational and rotational forces but provides less resistance against compressive forces on the anterior column [23]. The use of polyaxial screws and semi-rigid rods ultimately allows a reduction in compression with the standing position. Lindtner et al. showed a significant reduction in lordotic angulation from 8.3° preoperatively to 0.7° at 3 weeks postoperative in the percutaneous group with less rigid rods and early mobilization [11]. Our series confirms this observation, showing an improvement in the lordotic malalignment in the first 6 months (Figures 2, 4, 5).

In our series, no implant failure has occurred. In the literature, this rate can be as high as 15% [18] (Table 4). To overcome this problem, we extensively employed cemented screws and utilized semi-rigid rods to reduce stress at the bone-implant interface. Lindtner et al. found no implant loosening in the group operated with semi-rigid rods despite a lower rate of cementing [11]. In contrast to the existing literature, a four levels fixation appears to be sufficient, especially with the widespread use of cemented screws [19].

More than two-thirds of our patients experienced at least one complication, which is lower than rates reported in similar studies combining open and percutaneous surgeries (84% for Caron et al. [6] and 85.7% for Westerveld et al. [7]).

The lower proportion of percutaneous surgeries in these studies may explain this difference. Percutaneous series showed fewer complications, with some studies reporting no (Bredin et al. [15]) or few complications (9.1% for Trungu et al. [24]). In our cohort, the overall complication rate was 1.5 times higher in the open surgery group (88.2%) compared to the percutaneous surgery group (55.2%), with no surgical complications observed following percutaneous surgeries. The infection rate following open osteosynthesis was 17.6%, comparable to findings from Backhaus et al. (14%) [18]. Percutaneous surgery offers several advantages in ASD cases, including reduced blood loss, transfusions, operating time, complications, and hospitalization duration [12, 20]. The increasing use of intraoperative CT scans may limit the need for open surgeries in cases of neurological compression.

In ASD patients with spinal fractures, surgery reduces the mortality rate to 23% compared to 51% for non-operated patients [6]. The mortality observed in our study was in line with the literature (Table 4). Little difference was found between AS (17.7%) and DISH (20%) [25].

In our series, age was a significant predictor of mortality. Some authors have highlighted that being over 70 years of age was a major risk factor for mortality [6, 21], by the greater number of co-morbidities [6]. Indeed, the mortality rate in the age group over 80 years was 28.6% and the mean age in our population (79.3 years) was greater than other studies with better mortality rates [15] (Table 4).

Associated trauma or epidural hematoma increased the risk of dying by more than 20 times (OR = 21.6, *p* = 0.001). Unfortunately, these parameters cannot be changed on admission, but this result can be used to properly inform patients and their families. Complications were also significantly correlated with mortality (*p* = 0.041). Pneumonia is known to be associated with increased in-hospital mortality [21]. As severe pulmonary complications are among the most frequent, special care must be taken in their management.

Unlike the series by Okada et al. [16], our study did not show any impact of surgical technique on mortality. Surprisingly, the delay from trauma to surgery does not seem to have an influence on mortality in spite of being bedridden for long time during this period.

In conclusion, isolated posterior stabilization in the treatment of thoracolumbar spine fractures in elderly ASD patients is a safe technique promoting autonomy preservation, and high radiological bony healing with acceptable complication and mortality rates. The persistent anterior gap is partially reduced when the spine is loaded and does not seem to require an anterior procedure, thus decreasing complications. Percutaneous surgery should be the technique of choice to reduce surgical complications.

## Data Availability

Data are available on request from the authors.
